# Drug Repurposing in the Treatment of Traumatic Brain Injury

**DOI:** 10.3389/fnins.2021.635483

**Published:** 2021-03-23

**Authors:** Michael K. Ghiam, Shrey D. Patel, Alan Hoffer, Warren R. Selman, Barry J. Hoffer, Michael E. Hoffer

**Affiliations:** ^1^Department of Otolaryngology, University of Miami Miller School of Medicine, Miami, FL, United States; ^2^University of Miami Miller School of Medicine, Miami, FL, United States; ^3^Department of Neurological Surgery, Case Western Reserve University School of Medicine, Cleveland, OH, United States; ^4^Department of Neurological Surgery, University of Miami Miller School of Medicine, Miami, FL, United States

**Keywords:** traumatic brain injury, concussion, N-acetyl cysteine, minocycline, phenserine

## Abstract

Traumatic brain injury (TBI) is the most common cause of morbidity among trauma patients; however, an effective pharmacological treatment has not yet been approved. Individuals with TBI are at greater risk of developing neurological illnesses such as Alzheimer’s disease (AD) and Parkinson’s disease (PD). The approval process for treatments can be accelerated by repurposing known drugs to treat the growing number of patients with TBI. This review focuses on the repurposing of N-acetyl cysteine (NAC), a drug currently approved to treat hepatotoxic overdose of acetaminophen. NAC also has antioxidant and anti-inflammatory properties that may be suitable for use in therapeutic treatments for TBI. Minocycline (MINO), a tetracycline antibiotic, has been shown to be effective in combination with NAC in preventing oligodendrocyte damage. (−)-phenserine (PHEN), an anti-acetylcholinesterase agent with additional non-cholinergic neuroprotective/neurotrophic properties initially developed to treat AD, has demonstrated efficacy in treating TBI. Recent literature indicates that NAC, MINO, and PHEN may serve as worthwhile repositioned therapeutics in treating TBI.

## Background

Traumatic brain injury (TBI) is a major public health issue with 69 million cases globally each year and plays a role in approximately one-third of all injury-related deaths in the United States (Faul et al., 2010; Dewan et al., 2018). North America has the highest reported incidence of TBI in the world with 1299 cases per 100,000 individuals at an estimated cost of $3.84 billion (Faul et al., 2007, 2010; Dewan et al., 2018). TBI cases are commonly classified as: *mild* (awake and oriented); *moderate* (significantly confused but able to follow commands); and *severe* (prolonged impaired consciousness and inability to follow commands) based on parameters of the Glasgow Coma Scale (Jennett et al., 1981). Most cases are mild in severity, allowing for the opportunity to recover from initial symptoms. However, despite initial recovery, patients remain at risk of developing late secondary neurodegenerative disorders such as Alzheimer’s disease (AD) (e.g., memory loss) and Parkinson’s disease (PD) (e.g., tremor and shuffling gait) (Goldman et al., 2006; Tagliaferri et al., 2006; Chauhan, 2014; Djordjevic et al., 2016; Mendez, 2017). Drugs utilized in treating TBI may work to protect brain areas that are at increased risk in the acute and delayed setting (Du et al., 2016). The mechanisms of injury following cortical impact involve neuroinflammatory pathways, apoptotic cell death, and glutamic toxicity (Ray et al., 2002). The multifaceted and undefined nature of TBI makes it difficult, time consuming, and expensive to develop novel treatment strategies. Drug repurposing can provide a fast-tracked route toward developing effective therapies for TBI. Several drugs under investigation include N-acetyl cysteine (NAC), minocycline (MINO), (−)-phenserine, progesterone, propranolol, and valproic acid (Eakin et al., 2014; Xiong et al., 2015; Hoffer et al., 2017). NAC and MINO have been approved by Federal and Drug Administration for the treatment of acetaminophen induced hepatotoxicity and bacterial infections, respectively (Adminstration, 1971; Prescott et al., 1979).

## Pathophysiology of Traumatic Brain Injury

Primary TBI occurs as a result of physical trauma to the head, causing neuronal damage, and neuroinflammatory events. The manipulation of neuronal membranes dysregulates ion flow. Neuroinflammation is induced by increased microglial activity and depletion of mitochondrial glutathione (GSH), a key antioxidant compound. GSH reduction combined with increased intracellular calcium ion induces mitochondrial dysfunction leading to caspase activation and eventual apoptosis (Xiong et al., 1999; Pearn et al., 2017). Secondary, or delayed, injury following TBI is believed to occur due to multiple physiologic processes including free radical injury, inflammation, and glutamatergic excitotoxicity (Lenzlinger et al., 2001; Morganti-Kossmann et al., 2002; Yi and Hazell, 2006; O’Connell and Littleton-Kearney, 2013).

## Current Therapies Under Investigation

### N-Acetyl Cysteine

N-acetyl cysteine has been shown to have significant neuroprotective effects in various animal models, particularly in ameliorating the effects of secondary neuronal injury as a result of TBI. Experimental rat models have confirmed the beneficial antioxidant properties of NAC when used to treat brain injury. NAC acts by upregulating the level of GSH, a combination of L-glutamic acid, L-cysteine, and glycine, within the brain. Administration of NAC maintains high GSH levels in the brain which acts as a free radical scavenger and as an antioxidant itself (Ellis et al., 1991; Xiong et al., 1999; Chen et al., 2008). The underlying pathophysiologic processes resulting from TBI lead to increased risk of neurodegenerative illnesses such as AD and PD (Ikonomovic et al., 2004; Acosta et al., 2013; Franzblau et al., 2013; Chauhan, 2014; Pearn et al., 2017; Gardner et al., 2018). NAC provides a source of cysteine, a precursor to GSH, which can be used to alleviate reactive oxygen species (ROS)-mediated complex I damage in mitochondria of the substantia nigra in PD (Jha et al., 2000; Martínez Banaclocha, 2000; Banaclocha, 2001). NAC also reduces tau and beta-amyloid deposition and acts as an anti-inflammatory agent in treating AD via the upregulation of GSH, displaying its efficacy in not only treating TBI itself but also subsequent neurodegenerative conditions that are associated with TBI in rat and mice models (Tucker et al., 2005; Acosta et al., 2013; Franzblau et al., 2013; Joy et al., 2018; Tardiolo et al., 2018). A double-blind placebo-controlled human trial was used to evaluate efficacy of NAC in patients with blast-induced mild TBI (Hoffer et al., 2013). The treatment group received 2 g of NAC twice daily for the first 4 days followed by 1.5 g of NAC twice daily for 3 days. All patients were evaluated for dizziness, headache, hearing loss, memory loss, sleep disturbances, and neurocognitive dysfunction following 7 days of treatment. Significant improvement (*p* < 0.01) with regards to these symptoms were seen 7 days post-treatment in those receiving NAC within 24 h of injury. Additionally, the treatment group had 86% chance of recovery. Outcomes from this study warrant further investigation on long-term outcomes of NAC treatment in TBI (Table 1).

**TABLE 1:** Summary of human trials for repurposed drugs in TBI.

	Reference (study design)	*N* (% male)	Mean age (range)	Control	Dose	Outcome measures	Findings
N-acetyl cysteine	Hoffer et al., 2013 (phase III)	81 (99%)	22 years (18–43)	Placebo	2 g BID for 4 days 1.5 g BID for 3 days	1. Controlled oral world association test, animal naming test, trail making test 2. Clinical assessment for hearing loss, headache, balance	1. Improved cognitive function 2. Amelioration of mild TBI symptoms
	Amen et al. 2011 (Prospective)	30 (100%)	NR	Self-matched	NR	1. SPECT image analysis 2. MicroCog Assessment of Cognitive Functioning	1. Increased cerebral blood flow 2. Improved cognitive function
Minocycline	Meythaler et al., 2019 (phase IIa)	15 (80%)	43 years (21–71)	Self-matched	Tier 1: 200 mg BID for 7 days Tier 2: 400 mg BID for 7 days	1. Disability rating scale 2. Neurocognitive outcome measures 3. Serum biomarkers, laboratory analysis	1. Trend toward improved DRS for higher dose 2. Safe for use in TBI at 2× dose recommended for infection
	Koulaeinejad et al., 2019 (phase III)	34 (88%)	42.5 years (18–73)	Placebo	100 mg BID for 7 days	1. S100B 2. NSE 3. GCS	1. Significant reduction S100B and NSE
	Scott et al., 2018 (cross sectional)	15 (87%)	42.3 years (23–61)	Control (no treatment)	100 mg BID for 12 weeks	1. PET 2. MRI 3. Plasma axonal protein NFL	1. Reduced chronic microglial activation 2. Increased plasma NFL 3. Microglial activation has reparative effects in late stage TBI

*TBI, traumatic brain injury; BID, twice daily; NR, not recorded; SPECT, single photon emission computed tomography; DRS, disability rating scale; NSE, neuron specific enolase; GCS, Glasgow Coma Scale; PET, positron emission tomography; MRI, magnetic resonance imaging; NFL, neurofilament light.*

The antioxidant properties of NAC make it particularly useful in treating dysfunction resulting from TBI by mitigating the damaged mitochondrial production of ROS. However, low bioavailability in the brain has created interest in N-acetyl cysteine amide (NACA) a form of NAC with higher cellular permeability. Recent animal studies have shown NACA to be significantly better than NAC in reducing oxidative stress when treating TBI (Pandya et al., 2014).

Treatment with NACA in rats has also been shown to protect the blood-brain barrier (BBB) and maintain brain homeostasis by alleviating oxidative stress caused by blast overpressure induced TBI (Kawoos et al., 2019). NACA promoted an antioxidant effect via activation of the nuclear factor erythroid 2-related factor 2 (Nrf2) – antioxidant response element (ARE) pathway. NACA also displayed antiapoptotic properties following TBI. Both antioxidant and antiapoptotic effects are induced by modulation of the ubiquitin protease system via activation of the Nrf2-ARE pathway (Ding et al., 2017; Zhou et al., 2018). It is important to note that these studies utilized blast overpressure techniques. Additional studies investigating NACA using “true” blast models as described by Rubovitch et al. (2011) rather than a “blast tube” may provide further insight into the protective effects of NACA.

### Minocycline

Minocycline (MINO), a tetracycline antibiotic, has been shown to offer neuroprotective properties on its own and in combination with NAC and NACA. Rat models have supported the efficacy of MINO in treating neurological impairment arising from TBI (Haber et al., 2018; Zhang et al., 2020). In a mild controlled cortical impact model, MINO + NAC improved memory and cognition in rats and repaired white matter damage by protecting oligodendrocytes (Haber et al., 2018). In a controlled head injury (CHI) model of TBI, loss of oligodendrocytes was also observed. MINO + NAC provided protection against oligodendrocyte apoptosis during days 2–14 when dosed at 12 h post-TBI. MINO alone did not protect against initial oligodendrocyte damage when dosed 12 h post-TBI; however, full recovery was seen on day 14, suggesting that MINO alone operates via a different mechanism than MINO + NAC (Sangobowale et al., 2018). MINO alone acts by temporarily inhibiting microglial activation and reducing TBI-induced locomotor activity leading to improved long-term outcomes post-TBI (Homsi et al., 2010). Despite MINO proving to be safe in treating TBI in phase 1 trials, a human study on 15 patients greater than 6 months post moderate/severe TBI, receiving 100 mg MINO two times daily, indicated inhibition of chronic microglial activation which may have reparative effects but ultimately resulted in increased neurodegeneration indicated by increased levels of plasma neurofilament light chain (Scott et al., 2018; Meythaler et al., 2019). Though MINO alone may not be efficacious in treating TBI, human trials involving MINO and NAC together may be warranted.

### (−)-Phenserine

Phenserine is an anti-acetylcholinesterase agent with additional non-cholinergic properties that was originally developed to treat AD (Greig et al., 1995). However, PHEN has the potential to be repurposed for treating TBI. PHEN anti-acetylcholinesterase activity has been shown to reduce neuroinflammation, alleviate amyloid deposition, and prevent apoptosis as well as mitigate multiple different mechanisms of secondary injury (Kadir et al., 2008; Poole and Agrawal, 2008; Hsueh et al., 2019; Lecca et al., 2019).

A study by Lecca et al. (2019) investigated the effects of PHEN (5 mg/kg) in mitigating apoptosis and neuroinflammation in both wild-type (WT) and amyloid-precursor protein (APP)/presenilin 1 (PSEN1) expressing AD mice (APP/PS1 mice) when exposed to mild TBI (mTBI). PHEN was found to be well tolerated in exposed mice. Exposure to mTBI resulted in increased size of microglial cell bodies and increased production of IB1A1 and TNF-α. PHEN reduced inflammation by decreasing the production of IB1A1 and TNF-α in microglial cells in a dose-dependent manner after exposure to mTBI in both WT and APP/PS1 mice. This anti-inflammatory action was observed in both hippocampal and cortical areas in WT and APP/PS1 mice. An increase in glial fibrillary acidic protein IR (GFAP IR), an astrocyte marker, was also noted in hippocampal in cortical areas. PHEN was shown to fully reverse the increase in GFAP IR in both WT and APP/PS1 mouse models (Lecca et al., 2019). PHEN has demonstrated both anti-apoptotic effects through upregulation of Bcl-2 and BDNF expression while also decreasing pro-apoptotic factors such as caspase-3, APP, and GFAP (Chang et al., 2017). Similar data was also seen following controlled cortical impact injury in moderate TBI. In addition to treating cell death, PHEN has also had positive effects on reducing intracranial pressure, measured with lateral ventricle size, and contusion volume. Treatment of 2.5 mg/kg twice daily for 5 days post controlled cortical impact injury in mice reduced both contusion volume and intracranial pressure (Hsueh et al., 2019).

Phenserine administration post mTBI not only alleviates primary cellular damage but also shows efficacy in treating secondary TBI syndromes such as AD (Ikonomovic et al., 2004; Acosta et al., 2013; Franzblau et al., 2013; Chauhan, 2014). Treatment with PHEN in WT mice exposed to mTBI displayed significant dose-dependent recovery of both visual and spatial memory (Lecca et al., 2019). Levels of APP and alpha-synuclein, which are present in AD and PD, respectively, were decreased by treatment of PHEN (Marutle et al., 2007; Mikkilineni et al., 2012; Chang et al., 2017).

While many studies have demonstrated the protective effects of PHEN due to its anti-cholinergic activity, recent studies have also shown that many of the protective effects of PHEN maybe mediated by multiple non-cholinergic mechanism. A study by Tweedie et al. (2016) evaluated the non-cholinergic actions of PHEN on mTBI mice following a 2 days wash out when cholinergic actions were no longer present. PHEN dosed at 2.5 and 5.0 mg/kg twice daily for 5 days mitigated oxidative stress as measured by activity and protein levels of superoxide dismutase and glutathione peroxidase. Furthermore, PHEN was shown to reverse hippocampal gene expression associated with lipid peroxidation and development of AD in mTBI mice (Tweedie et al., 2016). Thus therapeutic benefits of PHEN in mitigating effects of TBI are likely related to both non-cholinergic features unique to PHEN as well as cholinergic properties shared by other anticholinesterases.

## Other Drugs Under Investigation

Many drugs are currently being evaluated as potential treatments for TBI. Though the exact mechanism is unknown, amantadine appears to act as an NMDA receptor antagonist and an indirect dopamine agonist. Placebo-controlled studies involving amantadine as a potential therapeutic agent have shown efficacy in accelerating recovery time and functional improvement following TBI (Meythaler et al., 2002; Giacino et al., 2012). Neurosteroids have also been considered to be repurposed to treat TBI. Progesterone, a neurosteroid, has been shown to promote neurogenesis by increasing the release of BDNF, NGF, and IGF (Stein and Sayeed, 2019). In a randomized controlled trial, progesterone administration lowered mortality 3 months post-TBI. Though it did not show significant benefit 6 months post-TBI, administration in combination with other drugs may support its utility (Pan et al., 2019). A summary of ongoing human clinical trials for the aforementioned repurposed drugs can be found in Table 2.

**TABLE 2:** Ongoing human clinical trials.

Clinical trial identifier	Status	Title	Study design	Drug	Intervention arms	Primary outcome measure
NCT03241732	Recruiting	PET-MRI and the effect of N-acetyl cysteine and anti-inflammatory diet in TBI	Non-randomized, crossover	NAC	1. Dietary arm 2. IV/PO arm 3. Control arm	FDG-PET to measure inflammation and oxidative damage in the brain
NCT04291066	Active, not recruiting	Prospective analysis of the use of N-acetyl cysteine and vitamins in the treatment of TBI in geriatric patients	Randomized, prospective	NAC and oral multivitamins	1. Experimental 2. Non-treatment (routine care)	Determine improvement in somatic, cognitive, and emotional post-concussion symptoms

*NAC, N-acetyl cysteine; TBI, traumatic brain injury; IV, intravenous; PO, per os; FDG-PET, fluorodeoxyglucose-positron emission tomography.*

## Conclusion

While effective pharmacological treatments have not yet been approved for the treatment of TBI, drug reproposing may help accelerate identification of effective pharmacological therapies. Limited human trials investigating NAC have shown promise and demonstrated neuroprotective effects in mTBI patients. Additional pre-clinical animal studies on NACA, MINO, and PHEN have also demonstrated efficacy in neuroprotection and mitigating delayed sequela from TBI. Future studies, particularly those involving human trials, are needed to elucidate the benefit of these reproposed drugs in mitigating the acute and delayed effects of TBI.

## Author Contributions

SP, MG, and MH: data analysis, manuscript preparation, and manuscript editing. WS, AH, and BH: manuscript preparation and manuscript editing. All authors contributed to the article and approved the submitted version.

## Conflict of Interest

The authors declare that the research was conducted in the absence of any commercial or financial relationships that could be construed as a potential conflict of interest.
